# Immunogenicity and safety of the diphtheria, pertussis, tetanus and inactivated poliovirus vaccine when co-administered with the human rotavirus vaccine (Rotarix) in healthy Japanese infants: a phase IV randomized study

**DOI:** 10.1080/21645515.2018.1564441

**Published:** 2019-02-20

**Authors:** Paul Gillard, Tsuyoshi Tamura, Haruo Kuroki, Yoshiyuki Morikawa, Leentje Moerman, Jose Parra, Yurina Kitamura, Kazuko Mihara, Arisa Okamasa

**Affiliations:** a Clinical Development, GSK, Wavre, Belgium; b Department of Pediatrics, Hashimoto Clinic, Tokyo, Japan; c Department of Pediatrics, Sotobo Children’s Clinic, Chiba, Japan; d Pediatrics, Child Support General Clinic, Tokyo Japan; e Global Regulatory Affairs, GSK, Wavre, Belgium; f Clinical Statistics, GSK, Wavre, Belgium; g Project Management, Japan Development Division, GSK, Tokyo, Japan; h Medicines Development Division, GSK, Tokyo, Japan

**Keywords:** DPT-IPV vaccine, rotavirus vaccine, co-administration, safety, immunogenicity, infants, Japan

## Abstract

Rotavirus infections have been reported to account for 40–50% of all hospitalized acute gastroenteritis cases in young children (<5 years) in Japan. Since 2011, Rotarix containing the live attenuated human rotavirus RIX4414 strain (HRV) has been licensed in Japan for infants. Vaccination against rotavirus is optional in Japan whereas administration of diphtheria, pertussis, tetanus, and inactivated poliovirus (DPT-IPV) vaccine is part of the national routine immunization program. In this open-label, randomized, controlled, multicenter study, we evaluated the immunogenicity and safety of the DPT-IPV vaccine (Squarekids) administered concomitantly or staggered with the liquid HRV (Rotarix) vaccine in healthy Japanese infants. A total of 292 infants aged 6–12 weeks were randomly assigned to receive DPT-IPV vaccine and HRV vaccine co-administered (n = 147) or staggered (n = 145). Immune responses to DPT-IPV vaccine were evaluated by measuring the post-vaccination serum antibody titers/concentrations to each antigen at one month following the third dose of DPT-IPV vaccine. Seroprotection/seropositivity against each of the diphtheria, pertussis (pertussis toxin and filamentous hemagglutinin), tetanus, and poliovirus type 1, 2 and 3 antigens was 92.8% or higher in both groups. In terms of immunogenicity, DPT-IPV vaccine co-administered with HRV vaccine was shown to be non-inferior to DPT-IPV vaccine with a staggered administration. The safety profile was comparable in the two vaccine groups with no vaccine-related serious adverse events, no deaths and no cases of intussusception. These results support co-administration of HRV vaccine with DPT-IPV vaccine in Japan.

ClinicalTrials.gov NCT02907216

## Introduction

By the age of 5 years, nearly every child worldwide will have suffered one or more rotavirus gastroenteritis (RVGE) episodes. The World Health Organization (WHO) estimates that globally 215,000 child deaths due to rotavirus infection occurred in 2013.^1-3^ More than 90% of these deaths were reported in low- and middle-income countries, especially in Africa and Asia due to lack of adequate access to health care.^2-4^ In developed countries, the burden of RVGE is also substantial as it leads to a high load for parental care and, in some instances, need for hospitalization. In Japan, a substantial disease burden of hospitalizations for acute gastroenteritis (AGE) of children younger than 5 years has been observed, with annual incidence rates up to 17.6 per 1,000 children.^5^ Rotavirus infections accounted for 40–50% of all hospitalizations for AGE.^6,7^

The WHO considers rotavirus vaccination as an effective measure to prevent RVGE and recommends it to be done as soon as possible from 6 weeks of age, if possible along with the diphtheria-pertussis-tetanus (DPT) vaccination to ensure induction of protection prior to the peak incidence of natural rotavirus infection.^2^ Rotavirus vaccines are recommended to be part of all national immunization programs, particularly in countries with high RVGE-associated fatality rates.^2^ A study from Mexico conducted 2 to 3 years after the introduction of rotavirus vaccine showed a 35% reduction in diarrhea-related deaths in children <5 years of age, suggesting a clear benefit from rotavirus vaccination.^8^

Two live attenuated rotavirus vaccines are licensed in Japan for infants: the human rotavirus (HRV) vaccine (Rotarix, GSK) was licensed in 2011 and the human-bovine reassortant rotavirus (HBRV) vaccine (RotaTeq, Merck & Co) in 2012. Both vaccines were studied in the Japanese population and were shown to be efficacious with an acceptable safety and immunogenicity profile. For HRV vaccine, a phase III, randomized, double-blind study conducted in Japan showed that the vaccine when administered in infants aged 6–14 weeks as a 2-dose (0, 1-month) schedule, led to a significant reduction of medical interventions by 79.3% (for any RVGE) and 91.6% (for severe RVGE) from two weeks post- Dose 2 until two years of age.^9^ A similar efficacy was observed when 3 doses of HBRV vaccine were administered in healthy Japanese infants, aged 6–12 weeks, preventing any severity and severe RVGE by 74.5% and 100%, respectively.^10^

Although the rotavirus vaccination for Japanese infants may be cost-effective by reducing the burden of RVGE and the use of healthcare resources,^11,12^ it is not part of the national routine immunization program whereas the combined DPT- inactivated poliovirus (DPT-IPV) vaccine is.^13^ Concomitant administration of HBRV vaccine with a DPT-IPV vaccine (Tetrabik, BIKEN, Osaka, Japan) was investigated and did not show any interaction in terms of immunogenicity and safety.^13^

Another DPT-IPV vaccine manufactured in Japan (Squarekids, Kitasato Daiichi Sankyo Vaccine Co., Ltd.), differs in its composition from the vaccine previously investigated. The purpose of the present study was to assess the immunogenicity and safety of DPT-IPV vaccine Squarekids when co-administered with HRV vaccine compared to staggered administration.

## Results

### Participant accounting and demographics

**Figure 1.**
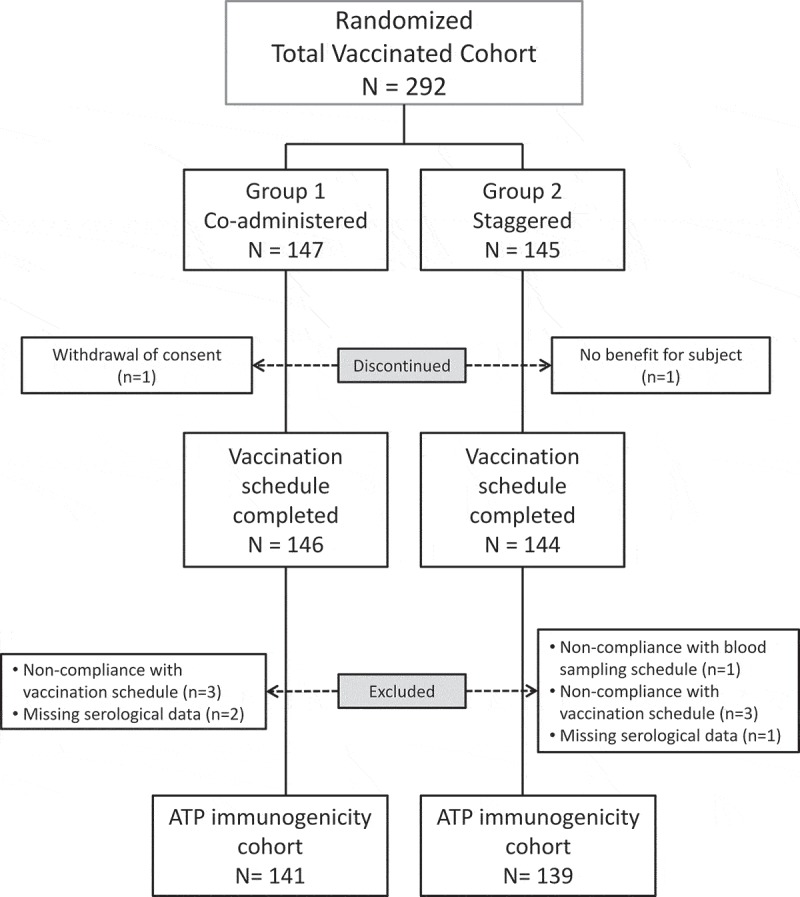
Study Flow. ATP, according-to-protocol cohort; N, number of individuals in the cohorts; n, number of individuals discontinued or excluded

The study was conducted between September 16, 2016 and May 29, 2017. A total of 292 healthy Japanese infants were enrolled and randomized in a 1:1 ratio to either Group 1 (co-administered group) or Group 2 (staggered group) (Figure 1). The percentages of boys and girls enrolled in Group 1 were similar (51%/49%) whereas there were slightly more boys than girls in Group 2 (55.2%/44.8%) (Table 1). All other demographic characteristics of the subjects, their mean age in particular, were similar in the two groups.

**Table 1.** Demographics of subjects at baseline, total vaccinated cohort (TVC).

Characteristics		Group 1 Co-administered N = 147		Group 2 Staggered N = 145	
Gender (n, %)	Female	72	(49.0)	65	(44.8)
	Male	75	(51.0)	80	(55.2)
Geographical ancestry (n, %)	Asian – Japanese Heritage	147	(100)	145	(100)
Age at each vaccine dose (weeks)					
Dose 1 of HRV	Mean (SD)	9.5	(1.1)	9.4	(1.1)
Dose 2 of HRV	Mean (SD)	14.0	(1.1)	15.5	(1.3)
Dose 1 of DPT-IPV	N	147		144	
	Mean (SD)	14.0	(1.1)	13.9	(1.0)
Dose 2 of DPT-IPV	N	146		144	
	Mean (SD)	18.5	(1.2)	20.0	(1.4)
Dose 3 of DPT-IPV	N	146		144	
	Mean (SD)	23.7	(1.8)	25.0	(1.9)
Weight (kg)	Mean (SD)	5.6	(0.7)	5.5	(0.7)
BMI (kg/m^2^)	Mean (SD)	16.5	(1.5)	16.6	(1.6)
Gestational age (weeks)	Mean (SD)	39.1	(1.1)	38.8	(1.1)

N/n = number of subjects; BMI: Body mass index; DPT-IPV, diphtheria-pertussis-tetanus and inactivated polio vaccine; HRV, human rotavirus vaccine; SD: Standard deviation

All but 2 subjects received all study doses. Ten additional subjects (5 in each vaccine administration group) were excluded from the according to protocol (ATP) analysis, primarily because of non-compliance with the vaccination schedule. Overall, 141 subjects in Group 1 and 139 subjects in Group 2 were included in the ATP cohort for immunogenicity analysis, and 147 subjects in Group 1 and 145 subjects in Group 2 were included in the total vaccinated cohort (TVC) for the safety analysis.

### Immunogenicity

#### Immunogenicity of the DPT-IPV vaccine

Assessed one month after the third dose of the DPT-IPV vaccine, at least 92.8% of the infants in the ATP cohort in both groups achieved seroprotection antibody concentrations/titers against diphtheria, tetanus and poliovirus types 1, 2, 3 and were seropositive for both pertussis antigens, i.e. pertussis toxin (PT) and filamentous hemagglutinin (FHA) (Table 2).

**Table 2.** Seroprotection rates (SPR) for diphtheria toxin, tetanus toxin, and polio virus type 1, 2 and 3 and seropositivity rates for pertussis (PT, FHA) for co-administered and staggered groups at one month following the third dose of DPT-IPV vaccine (ATP cohort for immunogenicity).

		Group 1 Co-administered		Group 2 Staggered		Estimated difference (Co-administered minus Staggered)		
Antigens/toxin	Threshold levels	N	SPR % (n/N)	N	SPR % (n/N)	%	95% CI	
Diphtheria Toxin	≥0.1 IU/mL	141	100	137	100	0.00	−2.66	2.74
Tetanus Toxin	≥0.1 IU/mL	141	98.6	138	99.3	−0.69	−4.39	2.71
Pertussis FHA	≥10 IU/mL	141	100	138	100	0.00	−2.66	2.72
Pertussis PT	≥10 IU/mL	141	95.7	138	92.8	2.99	−2.70	9.12
Poliovirus Type 1	NAT ≥ 8 ED_50_	140	100	137	100	0.00	−2.68	2.74
Poliovirus Type 2	NAT ≥ 8 ED_50_	128	100	127	100	0.00	−2.92	2.95
Poliovirus Type 3	NAT ≥ 8 ED_50_	132	100	123	99.2	0.81	−2.04	4.47

ATP, according to protocol; CI, Confidence interval; DPT-IPV, diphtheria-pertussis-tetanus and inactivated polio vaccine; ED_50_, 50% effective dose; FHA, pertussis filamentous hemagglutinin; n, Number of subjects who achieved the seroprotection/seropositivity threshold; N, Number of subjects in the ATP cohort for immunogenicity analyses; NAT, Neutralizing antibody titers; PT, pertussis toxin; SPR: Seroprotection/seropositivity rate.

Assessed at the same time point, the geometric mean titers (GMTs) and/or geometric mean concentrations (GMCs) of each antibody for diphtheria, tetanus, pertussis PT and FHA, and poliovirus types 1, 2 and 3 were similar in the two vaccination groups (Table 3).

**Table 3.** Geometric mean titers (GMTs) and concentrations (GMCs) for diphtheria toxin, tetanus toxin, pertussis (PT, FHA), and polio virus type 1, 2 and 3 for co-administered and staggered groups at one month following the third dose of DPT-IPV vaccine (ATP cohort for immunogenicity).

	Group 1 Co-administered				Group 2 Staggered			
Antigens/toxin	N	GMC or GMT	95% CI		N	GMC or GMT	95% CI	
Diphtheria Toxin	141	5.4	4.9	6.0	137	6.0	5.5	6.6
Tetanus Toxin	141	1.6	1.3	2.0	138	2.0	1.7	2.4
Pertussis FHA	141	83.7	74.8	93.6	138	97.2	86.7	109.0
Pertussis PT	141	31.5	28.4	34.9	138	31.5	27.9	35.6
Poliovirus Type 1	140	404.7	341.4	479.8	137	427.9	359.3	509.6
Poliovirus Type 2	128	371.0	307.0	448.5	127	470.6	388.3	570.3
Poliovirus Type 3	132	436.3	365.6	520.7	123	409.8	330.8	507.5

ATP, according to protocol; CI, Confidence interval; DPT-IPV, diphtheria-pertussis-tetanus and inactivated polio vaccine; ED_50_, 50% effective dose; FHA, pertussis filamentous hemagglutinin; GMC, Geometric mean concentration; GMT, Geometric mean titer; N, Number of subjects in the ATP cohort for immunogenicity analyses; PT, pertussis toxin.

#### Immunogenicity of the HRV vaccine

One month after the second dose of HRV vaccine, 92.8% (95% CI: 83.9–97.6) of the subjects in the co-administered group and 92.5% (95% CI: 83.4–97.5) in the staggered group had seropositive anti-rotavirus immunoglobulin A (IgA) concentrations (≥20 U/mL). The anti-rotavirus GMC in infants in the co-administered group was 350.1 U/mL (95% CI: 223.3–548.8) compared to 362.5 U/mL (95% CI: 251.0–523.5) in the staggered group.

## Safety

The frequency of solicited and unsolicited, general and local adverse events (AEs) reported during the 8-day post-vaccination period following Dose 1 of the DPT-IPV vaccine was similar in both vaccine groups: 89.1% of the subjects (131/147) in the co-administered group vs. 86.1% of those (124/144) in the staggered group. A slightly higher incidence of general AEs was observed in the co-administered group as compared to the staggered group: 78.2% (115/147) and 70.1% (101/144), respectively; however, the reactogenicity profile appeared to be similar for each of the 4 solicited general symptoms (Table 4).Overall, 87.1% of the subjects (128/147) in the co-administered group and 85.5% of the subjects (124/145) in the staggered group experienced at least one adverse event (AE) during the 8-day post-vaccination period following HRV vaccinations (Table 4). The observed incidences of ‘loss of appetite’, ‘fever’ and ‘irritability/fussiness’ after the second HRV dose when co-administered were higher compared to staggered administration probably confounded by the reactogenicity of the DPT-IPV vaccine.

**Table 4.** Summary of adverse events, total vaccinated cohort (TVC).

		Group 1 Co-administered		Group 2 Staggered	
		n/N	%	n/N	%
AEs (solicited and unsolicited) during the 8-day post-vaccination period following HRV vaccine	Dose 1	102/147	69.4	104/145	71.7
	Dose 2	115/147	78.2	89/145	61.4
	Overall/subject	128/147	87.1	124/145	85.5
AEs (solicited and unsolicited) during the 8-day post-vaccination period following DPT-IPV vaccine	Dose 1	131/147	89.1	124/144	86.1
Unsolicited AEs within the 31-day post-vaccination period following HRV vaccine	Any dose	88/147	59.9	81/145	55.9
Unsolicited AEs within the 31-day post-vaccination period following first dose of DPT-IPV vaccine	Dose 1	65/147	44.2	59/144	41.0
At least one SAE		4/147	2.7	5/145	3.4
At least one vaccine-related SAE		0/147	0.0	0/145	0.0
Death		0/147	0.0	0/145	0.0
Discontinuation due to AEs		0/147	0.0	0/145	0.0

AE, Adverse event; DPT-IPV, diphtheria-pertussis-tetanus and inactivated polio vaccine; HRV, Human rotavirus vaccine; N, Number of subjects; n: number of subjects reporting an AE; %: percentage of subjects reporting an AE; SAE, Serious adverse event; TVC: total vaccinated cohort.

Similar proportions of subjects had at least one unsolicited AE during the 31-day (Days 0–30) post-vaccination period. After any HRV dose, these percentages were 59.9% (88/147) and 55.9% (81/145) in the co-administered and staggered group, respectively. After the first dose of DPT-IPV, the corresponding percentages were 44.2% (65/147) and 41.0% (59/144).

Serious adverse events (SAEs) were reported in 4 (2.7%) subjects in the co-administered group and in 5 (3.4%) in the staggered group. All subjects recovered from their SAEs and no SAE was considered by the investigator to be related to the study vaccines. By system organ class, the most common SAEs were infections and infestations (n = 7). There were neither reports of intussusception nor deaths during the study.

## Discussion

The DPT-IPV combined vaccine was introduced into the Japanese routine vaccination program on November 1, 2012. Although the high incidence of severe rotavirus disease leads to a substantial burden among Japanese children,^7^ vaccination against rotavirus is done on a voluntary basis.^14^ The first dose of the HRV vaccine is recommended to be administered during the age interval 6–14 weeks with a schedule of 2 vaccinations with HRV vaccine, or 3 vaccinations with HBRV vaccine. Since the routine vaccination program requires that children receive several vaccines around the same age, it is important to investigate whether concomitant administration of these 2 vaccines, DPT-IPV vaccine and HRV vaccine, could lead to interactions with regard to immunogenicity, safety and reactogenicity.

Although the available evidence suggests that rotavirus vaccine does not interfere with the immune response to these routine childhood vaccines,^9,15-19^ to our knowledge, this is the first study conducted in healthy Japanese infants aged 6–12 weeks to evaluate the immunogenicity and safety of co-administration of the DPT-IPV vaccine (Squarekids) and the oral liquid HRV vaccine compared to staggered administration. The immunogenicity results indicated that ≥ 95.7% of infants in the co-administered group and ≥ 92.8% in the staggered group were seroprotected for anti-D, anti-T and anti-polio 1, 2 and 3, and were seropositive for anti-PT and anti-FHA antibodies. The criteria for non-inferiority were reached for all antigens of the DPT-IPV components investigated in the study.

These findings are consistent with previous data observed on co-administration of HRV vaccine and DPT-IPV vaccine from the same manufacturer during the clinical development of the HRV vaccine.^15,20^ Although the immunologic mechanisms of protection to rotavirus disease are not fully understood, a positive serum anti-rotavirus IgA concentration ≥20 U/mL following vaccination appears to be a useful correlate of efficacy in clinical trials of HRV vaccine.^21-23^ We observed high seropositivity rates in both vaccine groups after 2 doses of HRV vaccine: 92.8% in the co-administered group and 92.5% in the staggered group. These anti-rotavirus IgA seropositivity rates were consistent with those obtained in a previous study showing a seroconversion rate of 85.3% (95% CI: 68.9–95%) one month post- Dose 2 of the HRV vaccine group in Japanese infants aged 6–14 weeks.^9^ The immunity against rotavirus induced by vaccination observed through anti-rotavirus IgA antibody responses after 2 doses of HRV vaccine was in line with that reported in Europe, Latin America, and Asia.^16,17,19^

Our study showed that the safety profile of co-administration or staggered administration of the two vaccines was similar. No case of intussusception was reported and there were no vaccine-related serious AEs or deaths reported during the study. This is in alignment with a previous report in the literature.^9^

However, this study has some limitations. This was an open-label study with slightly differing solicited symptom reporting which limits comparative assessments between groups. The specificities of the immunization schedule and the type of co-administered vaccines are differing from immunization practices out of Japan which impairs generalizability of the results. The results of this study should be interpreted with cautious as infants enrolled in the study may not be fully representative of the Japanese pediatric population as a whole.

**Figure 2.**
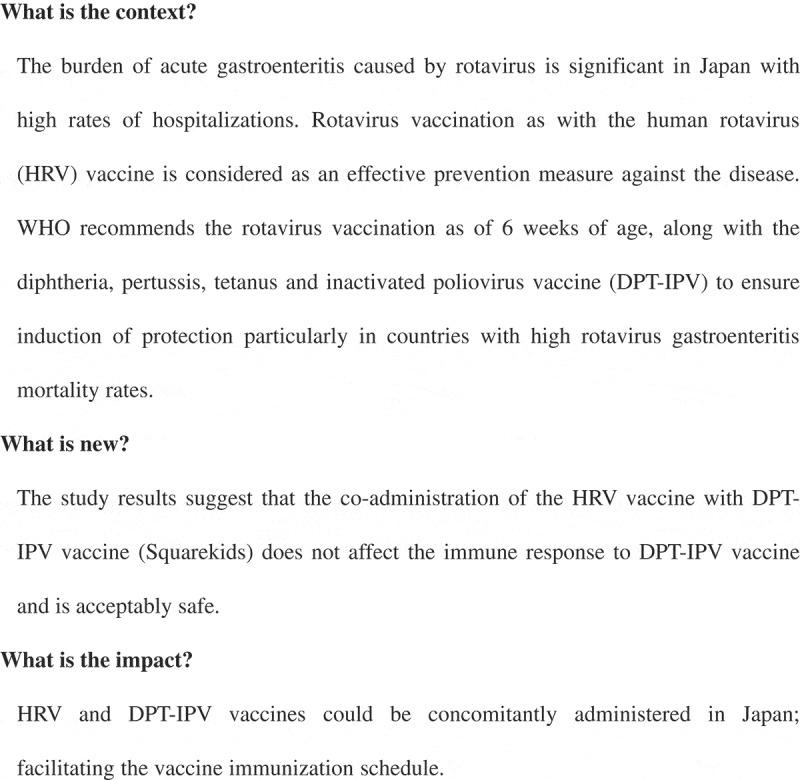
Focus on the patient.

In conclusion, our study shows that co-administration of DPT-IPV vaccine and HRV vaccine in Japanese infants did not impair the immune response to any of the following antigens in DPT-IPV: diphtheria, pertussis (PT, FHA), tetanus, and poliovirus type 1, 2, and 3. DPT-IPV vaccine and HRV vaccine were well tolerated in Japanese infants receiving the two vaccines concomitantly. These results support a co-administration of HRV vaccine with DPT-IPV vaccine in Japan. Figure 2 presents a summary of the context, outcomes, and impact of this study for healthcare providers.

## Patients and methods

### Study design

This was a phase IV, randomized, open-label, controlled, multicenter study with two experimental parallel arms (co-administered *vs*. staggered groups) conducted in 11 sites in Japan. The study aimed to assess whether the immunogenicity and safety of the diphtheria, pertussis (PT, FHA), tetanus and inactivated poliovirus (DPT-IPV) vaccine (Squarekids) was impaired when co-administered with GSK Biologicals’ oral live attenuated liquid HRV vaccine (Rotarix) compared to staggered administration. This study is registered with ClinicalTrials.gov, number NCT02907216.

The sample size was estimated in order to obtain at least 90% power to demonstrate the primary confirmatory objective of the study (Bonferroni adjustment of type II error).

A total sample size of 292 healthy infants 6–12 weeks of age were enrolled and randomized using a 1:1 ratio to one of the 2 groups.

**Figure 3.**
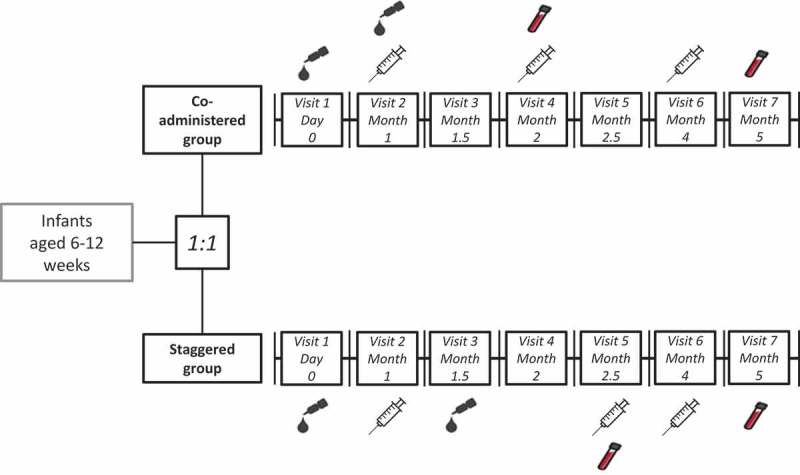
Vaccination schedule in the co-administered group and staggered group. 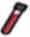
, blood sample; 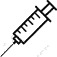 DPT-IPV vaccine, diphtheria-pertussis-tetanus and inactivated polio vaccine; 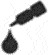 HRV vaccine: oral human rotavirus vaccine

Subjects in the co-administered group were administered the DPT-IPV vaccine according to a 3-, 4-, 6-months-of-age schedule and the HRV vaccine according to a 2-, 3-months-of-age schedule, in accordance with the recommended vaccination schedule in Japan. Co-administration of the study vaccines was performed only once, at Visit 2, when the infant was approximately 3 months old. Subjects in the staggered group were administered the DPT-IPV vaccine according to a 3-, 4.5-, 6-months-of-age schedule and the HRV vaccine according to a 2-, 3.5 months-of-age schedule. The duration of the study per subject was planned to be 5 months from Visit 1 (Day 0) to Visit 7 (Month 5). The vaccination schedules are presented in Figure 3.

The subjects could receive the routine vaccinations like pneumococcal conjugate vaccine, *Haemophilus influenzae* type B vaccine, Bacillus Calmette-Guérin vaccine, hepatitis B vaccine, meningococcal vaccine and inactivated influenza vaccine in accordance with clinical practice in Japan, at any time during the study, if administered at sites different from the sites used to administer the DPT-IPV vaccine. To exclude the influence of other pediatric vaccines on the immune responses, concomitant administration of all other pediatric vaccines was prohibited from 30 days before administration of the first dose of HRV vaccine until the study end (Visit 7).

Serum samples (5 mL) were collected at Visit 7, one month after administration of the last dose of DPT-IPV vaccine to measure the antibody response to DPT-IPV vaccine. A serum sample (2 mL) was collected at Visit 4 (co-administration group) or Visit 5 (staggered group) from a sub-cohort of subjects one month after the administration of Dose 2 of the HRV vaccine to measure the antibody response to HRV vaccine. The sub-cohorts for this assessment included approximately half the number of subjects in each study group.

### Study objectives

The primary study objective was to show that the immunogenicity to the antigens contained in DPT-IPV vaccine was not impaired by co-administration with the oral HRV vaccine. Secondary objectives were to assess the immunogenicity to all the antigens contained in the DPT-IPV vaccine in terms of GMCs/GMTs one month after the third dose of the DPT-IPV vaccine and to assess the immunogenicity of the HRV vaccine in terms of serum anti-rotavirus IgA antibody seropositivity and GMCs in a sub-cohort of subjects at one month after the second dose of the HRV vaccine. Safety of HRV vaccine and DPT-IPV vaccine were evaluated from the first administration of the study vaccines until the end of the study.

### Study population

Japanese male and female healthy infants aged between 6 and 12 weeks at the time of the first dose of HRV vaccine were eligible for the study. Exclusion criteria were checked at the start of the study and excluded child in care; receipt of any investigational or nonregistered drug during the 30 days preceding study entry and/or planned use during the entire study period; participation in another interventional clinical study at any time during the study; rotavirus, diphtheria, pertussis, tetanus, and/or poliomyelitis vaccination or disease; gastroenteritis during the 7 days preceding the HRV vaccine administration; a predisposition to and/or a history of intussusception; any confirmed or suspected immunosuppressive condition or immunodeficiency, a family history of immunodeficiency, or a history of severe combined immunodeficiency; major congenital defects or serious chronic illness; a known hypersensitivity to any of the HRV vaccine or DPT-IPV vaccine components, or to latex; a history of neurological disorders or seizures; acute disease and/or fever at the time of assessment for inclusion; chronic administration (defined as a total of >14 days) of immunosuppressants, other immune-modifying drugs, or prednisone (≥0.5 mg/kg/day or equivalent) since birth. Administration of immunoglobulins and/or any blood products since birth or planned during the study; administration of long-acting immune-modifying drugs at any time during the study were not allowed.

The study was conducted in accordance with the principles of good clinical practice, approved by the institutional review board of each participating site, and written informed consent was obtained from parent/legal guardian of each subject before study entry.

### Vaccine descriptions

HRV vaccine (Rotarix, GSK) is supplied in a 1.5 mL pre-filled oral applicator containing ≥10^6.0^ median cell culture infective dose of live-attenuated RIX4414 human rotavirus strain. Each dose of HRV vaccine was administered orally.

DPT-IPV vaccine (Squarekids, Kitasato Daiichi Sankyo Vaccine Co., Ltd) is supplied in a 0.5 mL pre-filled syringe. It is a tetravalent DPT-IPV combination vaccine that contains Salk inactivated polio vaccine. Each 0.5 mL dose of this vaccine contains ≥4 units of the *Bordetella pertussis* protective antigen, ≥14 international units (IU) of diphtheria toxoid, ≥9 IU of tetanus toxoid, 40 D-antigen units (DU) of inactivated poliovirus type 1, 8 DU of inactivated poliovirus type 2, and 32 DU of inactivated poliovirus type 3. The study administration consisted of 3 doses of 0.5 mL, each given by subcutaneous injection.

## Measurements

### Immunogenicity

After centrifugation, serum samples were kept at – 20°C or below until shipment. Serological assays for the determination of antibodies were performed by enzyme-linked immunosorbent assay or neutralization assay at GSK Biologicals’ laboratories (Belgium), using standardized and validated procedures. The primary endpoint was the immunogenicity with respect to the components of the DPT-IPV vaccine one month after administration of the third dose of the vaccine (Visit 7). The seroprotection rates for DPT-IPV vaccine were defined as ≥0.1 IU/mL for anti-diphtheria antibody concentrations, ≥0.1 IU/mL for anti-tetanus antibody concentrations, and ≥8 50% effective dose (ED_50_) for anti-poliovirus serotypes 1, 2 and 3 antibody titers. Regarding pertussis antigens, the seropositivity rates for DPT-IPV vaccine were defined as ≥10 IU/mL for anti-PT and anti-FHA antibody concentrations.

Anti-RV IgA antibody concentrations were measured using an in-house enzyme-linked immunosorbent assay (ELISA). Seropositivity was defined as anti-RV IgA antibody concentration greater than or equal to 20 U/mL.

### Safety

The safety assessment period started at the time of administration of the first vaccine dose and ended 5 months later, at Visit 7. The subject’s parents or legal representatives were asked to record axillary or tympanic body temperature and any solicited/unsolicited AEs on a vaccination diary card.

The time period for recording solicited AEs and body temperature was 8 days (Day 0–7) following each HRV vaccination and the first DPT-IPV vaccine dose. The time period for recording unsolicited AEs was 31 days (Day 0–30) after each HRV vaccine dose and the first dose of DPT-IPV vaccine. Solicited general AEs included fever, irritability/fussiness, diarrhea, vomiting, loss of appetite and cough/runny nose after HRV vaccine, and drowsiness, fever, irritability/fussiness and loss of appetite after DPT-IPV vaccine. It should be noted that the number of solicited general symptoms differed between the vaccines, which can impact the estimate of the incidence of all solicited symptoms in the co-administered group. Local AEs of pain, redness and swelling were solicited after DPT-IPV vaccine.

All SAEs (including those related to study participation or concurrent use of GSK medication/vaccine), causally-related AEs and AEs leading to withdrawal from the study were collected and recorded from the time of the first administration of study vaccines until the subjects’ discharge from the study.

### Statistical analysis

#### Immunogenicity

**Figure 4.**
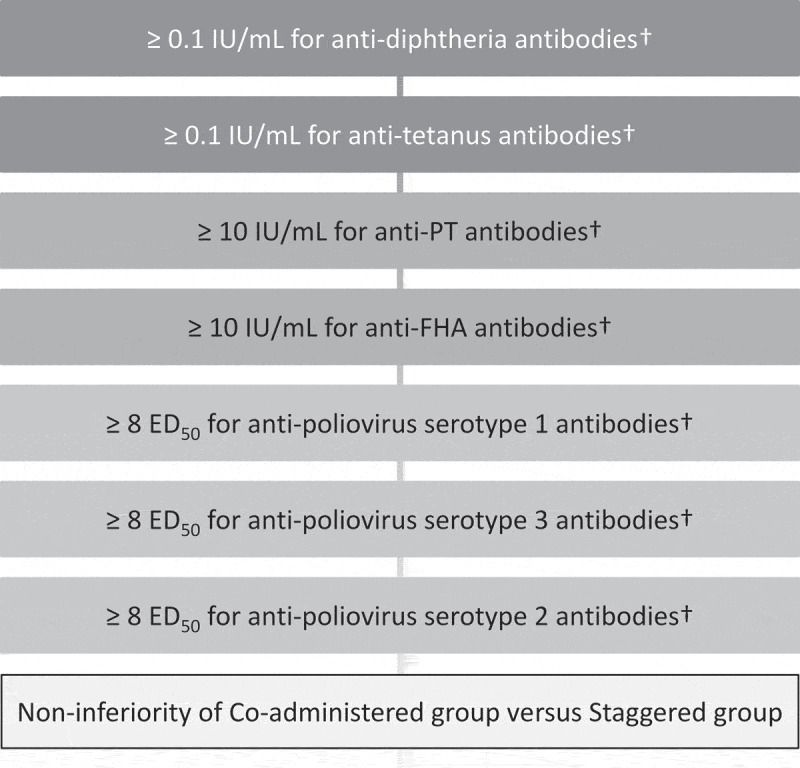
Hierarchical procedure to test the primary confirmatory objective. ED_50_, 50% effective dose; FHA, pertussis filamentous hemagglutinin; IU/mL, international unit per milliliter; PT, pertussis toxin. ^†^In terms of percentages of subjects with concentrations ≥10 IU/mL for anti-PT and anti-FHA, seroprotective concentrations for anti-D and anti-T or seroprotective titers for anti-IPV1, 2 and 3 at Visit 7 using a nominal alpha risk of 2.5%.ED_50_

The primary objective was to demonstrate non-inferiority in the percentage of subjects seroprotected for anti-diphtheria, anti-tetanus and anti-poliovirus 1, 2 and 3 and seropositive for anti-PT and anti-FHA when DPT-IPV vaccine was co-administered with HRV vaccine compared to staggered administration. Therefore, a composite of hypotheses was defined, one for each antigen to be tested hierarchically (Figure 4) to keep an overall type I error at 5%. The immunogenicity of the 3-dose regimen of DPT-IPV vaccine when co-administered with HRV vaccine (Group 1) was to be considered non-inferior to staggered administration (Group 2) if the lower limit of the 2-sided 95% CI of the between group difference of each of the DPT-IPV vaccine antigens (co-administered group minus staggered group) was above or equal to minus 10%. This criterion was based on clinical judgment and a previously published randomized trial evaluating vaccines with a comparable design.^13^

For each antigen, the difference in seroprotection and seropositivity rates between the co-administrated and staggered groups and its 95% standardized asymptotic CI were calculated using the Miettinen and Nurminen approach.^24^ Within groups, assessments consisted in estimation of seroprotection/seropositivity rates with exact two-sided 95% CIs^25^ and GMCs and GMTs were estimated with their 95% CIs.

The primary analysis of immunogenicity was performed on the ATP cohort, defined as eligible subjects who complied with the vaccination blood sampling schedules and whose concomitant medications and underlying medical conditions were not prohibited by the protocol. Subjects with any concomitant infection related to the vaccine that could influence the immune response were eliminated from the ATP cohort for immunogenicity.

#### Safety

The safety and reactogenicity were evaluated on the TVC that included all subjects with documented administration of at least one dose of the study vaccines. The percentage (with exact 95% CIs) of subjects experiencing at least one AE (any type), at least one local AE (solicited or unsolicited), at least one general AE (solicited or unsolicited) and at least one SAE were tabulated for both vaccination groups. Unsolicited symptoms were categorized from the verbatim reports by preferred term according to the latest version of the Medical Dictionary for Regulatory Activities classification.

## Data Availability

Anonymized individual participant data and study documents can be requested for further research from www.clinicalstudydatarequest.com

## Trademark statement

Rotarix is a trademark owned by the GSK group of companies.

RotaTeq is a trademark of Merck & Co, Inc.

Squarekids is a trademark of Kitasato Daiichi Sankyo Vaccine Co., Ltd.

Tetrabik is a trademark of BIKEN
